# Long-term clinical value of the anterolateral thigh perforator flap in the repair of limb soft tissue defects: A case series

**DOI:** 10.12669/pjms.41.12.12994

**Published:** 2025-12

**Authors:** Jirong Huang, Cuimei Rong, Lirong Hong

**Affiliations:** 1 Jirong Huang, Department of Burn and Plastic Surgery, People’s Hospital of Guigang City, Guangxi, Guigang, Guangxi Province 537100, P.R. China; 2 Cuimei Rong, Department of Oncology, People’s Hospital of Guigang City, Guangxi, Guigang, Guangxi Province 537100, P.R. China; 3 Lirong Hong, Department of Burn and Plastic Surgery, People’s Hospital of Guigang City, Guangxi, Guigang, Guangxi Province 537100, P.R. China

**Keywords:** anterolateral thigh, perforator flap, soft tissue, limbs

## Abstract

**Objective:**

To explore the long-term clinical value of the anterolateral thigh (ALT) perforator flap in the repair of limb soft tissue defects.

**Methodology:**

This retrospective analysis included clinical records of 62 patients with limb soft tissue defects who received ALT perforator flap treatment at Guigang People’s Hospital from January 2020 to October 2024. The defect area ranged from 8×6 cm to 28×15 cm. The arteries, veins and sensory nerves of the perforator flap were microanastomosed with the corresponding structures of the recipient area and the donor area was directly sutured in one stage. Patients were followed up eight months to five years after surgery.

**Results:**

Postoperative follow-up ranged from 8~60 months (average 32.9 ± 13.4 months); 59 cases of skin flaps survived completely, with a success rate of 95.2%. Three patients had reported complications: one case had localized necrosis at the edge and two cases had full-thickness necrosis, which healed after debridement and skin grafting. The skin flap donor site was directly sutured without the occurrence of fascial compartment syndrome. The microcirculation of the surviving skin flap was good, the color coordinated with the surrounding tissues and the texture was flexible. Three patients underwent secondary volume reduction plastic surgery due to the large volume of the reconstruction area, which affected their function. The postoperative contour was satisfactory.

**Conclusions:**

Long-term follow-up has confirmed that the ALT perforator flap can be used as a reconstruction treatment plan for soft tissue defects in limbs.

## INTRODUCTION

Soft tissue defects in limbs, caused by trauma, infectious lesions, radical resection of malignant tumors and refractory ulcers, often require reconstructive surgery.^1^ Various repair methods, such as adjacent flap transfer and distal pedicle flap transplantation, have shown inherent deficiencies in tissue acquisition, control of donor site complications and functional recovery.^1^-^3^ Adjacent skin flaps have a reliable blood supply, but cannot be used for repairing significant area defects. The distal pedicled skin flap involves a large amount of tissue, but requires secondary surgical resection of the donor site and long-term posture maintenance, which can lead to related complications in patients.^2^

Traditional reconstructive techniques, including adjacent skin flaps, pedicled flaps, and musculocutaneous flaps, often face several limitations. Adjacent flaps may lack sufficient tissue volume to cover large or complex defects.^3^ Pedicled flaps, while offering more extensive coverage, frequently require prolonged immobilization and pose risks of donor site morbidity and postoperative complications.^4^ Musculocutaneous flaps, although robust, can result in significant functional loss at the donor site. Furthermore, most of these techniques require multistage operations and may not allow for individualized flap design based on recipient site needs. In contrast, the anterolateral thigh (ALT) perforator flap has gained attention for its favorable vascular anatomy, long pedicle length (8–12 cm), and reliable microvascular anastomosis.^5^ It enables a substantial soft tissue harvest with minimal donor site morbidity and allows incorporation of sensory nerves to facilitate functional recovery. Increasing studies have demonstrated its advantages in achieving both aesthetic (color and texture match) and functional (sensory recovery) outcomes. However, long-term outcome data remain limited. This study aims to fill that gap by analyzing long-term follow-up data in a relatively large patient cohort.

Studies show that a perforating vascular skin flap can provide a relatively stable and complete blood supply to the flap during surgery and complete the blood supply to the lesion site while minimizing functional loss in the supply area.^2^-^5^ The ALT perforator flaps are associated with a reduced risk of donor-site functional loss and allow individualized design tailored to recipient-site characteristics; however, in some cases, residual bulk may still limit recipient-site function.^5^,^6^ The ALT perforator flap, first proposed in 1984, has become one of the commonly used skin flaps in reconstructive surgery, as it offers the advantages of anatomical stability, sufficient tissue supply and fewer complications in the distal flap supply area.^5^

The ALT flap uses the musculocutaneous or intermuscular septal perforating branch of the descending branch of the lateral circumflex femoral artery as its vascular pedicle, which allows for a flap size of 25 × 35 cm. The longest vascular pedicle can reach 8~12 cm and the vessel diameter ranges from 1.5 to 3.0 mm, which is conducive to microvascular anastomosis.^5^-^7^ However, due to a significant variability in the number, location and size of perforating vessels in the anterior lateral thigh, this region is considered anatomically uncertain.^7^ In recent years, studies have reported cutting and segmenting large wounds, thereby converting the width of the skin flap into length, which achieves direct closure of the skin flap donor area and effectively avoids damage to the second donor area.^7^,^8^ However, most studies have short follow-up times and a small number of cases. This study aimed to clarify the long-term clinical application value of the ALT perforator flap and provide a practical reference for clinical workers.

## METHODOLOGY

This retrospective analysis included data from 62 patients with soft tissue limb defects who received ALT perforator flap treatment at Guigang People’s Hospital from January 2020 to October 2024 and were followed up from eight months to five years post-surgery.

### Ethical approval:

This study was approved by the medical ethics committee of Guigang people’s hospital (No.: E2024-020-01; Date: 5^th^ May, 2024). Written informed consent was obtained from all patients or their legal guardians prior to treatment and data collection.

### Inclusion criteria:

- The recipient vessels were well conditioned and suitable for microvascular anastomosis.
- The anterolateral thigh skin of the donor site was intact and could be directly sutured.
- The follow-up time was ≥ eight months.
- The clinical data are complete.

### Exclusion criteria:

Severe cardiopulmonary insufficiency.
Coagulation dysfunction.
Active wound infection was not controlled.

### Collected outcomes:

- Basic information (age, gender, cause of injury, defect location and area).
- Operation-related information (flap area, vascular pedicle length and diameter, operation time).
- Postoperative outcome indicators (flap survival rate, type of complications, donor site healing, sensory function recovery level, secondary surgery).
- Long-term follow-up data (follow-up time, flap blood supply status, appearance satisfaction, functional recovery).

### Repair Surgery:

### Preoperative preparation:

Upon admission, immediate debridement was performed to remove contaminants and necrotic tissue, followed by external fixation and internal reduction of the fracture.

### Periodic repair:

If there were no exceptional circumstances, all repairs were attempted in one phase whenever possible. Alternatively, the repairs were done in stages.

### Skin flap design:

The maximum width of direct closure was determined using the skin retraction testing method and a multi-flap design was used beyond the range. According to the geometric shape of the wound and the distribution of perforating blood vessels, a multi-leaflet structure was formed.

### Skin flap harvesting and repair:

The perforating blood vessels were carefully dissected under a surgical microscope to ensure good blood supply before preparing the skin flap. The skin flap passed through the arteriovenous system and anastomosed end-to-end with the corresponding blood vessels in the recipient area. The sensory nerves were sutured microscopically with the cutaneous nerves in the recipient area. The supply area was closed directly using cosmetic surgical suturing techniques. The sensory function was evaluated according to the British Medical Research Council classification: S4, normal sensitivity; S3+, recovery of useful discriminatory sensitivity; S3, recovery of complete tactile sensitivity without dysesthesia and rough useful discrimination; S2, recovery of superficial painful and incomplete tactile sensitivity with hyperesthesia and/or dysesthesia; S1, recovery of deep painful sensitivity and S0, no sensitivity.

### Postoperative treatment:

Standard nursing procedures were followed after free tissue transplantation. Continuous follow-up after surgery was done to evaluate the blood circulation, sensory function recovery and donor site healing of the skin flap.

## RESULTS

As shown in Table-I, among the 62 patients included in the study, there were 42 males and 20 females, with an age range of 12~65 years (average age, 36.3 ± 14.4 years). There were 21 cases of foot injuries and 41 cases of upper limb injuries. Causes of injury were as follows: 18 cases of agricultural machinery twisting injuries, 35 cases of traffic injuries, four instances of mechanical compression injuries and five cases of heavy object injuries. The defect area after debridement was 8×6 cm to 28×15 cm.

**Table-I:** Basic Patient Information.

Information	Proportion
Age (years), mean±SD	36.3±14.4
Male, n(%)	42 (67.7)
***Defect location, n(%)***	
Foot defect	21 (33.9)
Upper limb defect	41 (66.1)
Defect area (cm)	8×6 - 28×15cm
***Cause of injury, n(%)***	
Agricultural machinery twisting injury	18 (29.0)
traffic accident	35 (56.5)
Machine squeezing	4 (6.5)
Heavy object injury	5 (8.0)

Out of 62 performed flaps, 59 achieved complete survival, with a success rate of 95.2% with no cases of infection. There was one case of a locally necrotic single-lobed skin flap and two instances of completely necrotic skin flaps that healed after re-debridement, skin grafting and other treatments. The specific causes of flap inactivation in the three cases were as follows: one case had marginal localized necrosis due to intraoperative perforator vessel injury and two cases were completely necrotic due to vasospasm and improper postoperative positioning. All wounds in the donor site were closed primarily with cosmetic suturing, and no compartment syndrome was observed.

During the follow-up period, which ranged from eight months to five years (with an average of 32.9 ± 13.4 months) after surgery, the surviving skin flap exhibited good blood circulation. It matched well in terms of color and texture with the recipient tissue. Three patients underwent secondary skin flap thin plastic surgery, which resulted in a slightly bulky appearance and satisfactory postoperative outcomes. Table-II shows the immediate and early postoperative complications. The sensory function was restored in all cases. Based on the British Medical Research Council classification, fifty-three cases (85.5%) were grade S3, six cases (9.7%) were grade S2 and three cases (4.8%) were grade S1. All survived flaps achieved different degrees of sensory function recovery (Table-III).

**Table-II:** Immediate and early postoperative complications (n=62).

Item	n (%)
***Flap survival***	
Complete survival	59 (95.2)
Local necrosis	1 (1.6)
Complete necrosis	2 (3.2)
***Infection***	
No infection	62 (100)
Superficial infection	0 (0.0)
Deep infection	0 (0.0)
***Donor site complications***	
Compartment syndrome	0 (0.0)
Donor site infection	0 (0.0)
Poor donor site healing	0 (0.0)
Successful first stage cosmetic suture	62 (100)

**Table-III:** Long-term follow-up results (n=59).

Item	n
***Blood circulation status of skin flap***	
Good blood supply	59 (100)
Average blood supply	0 (0.0)
Poor blood circulation	0 (0.0)
***Color matching***	
Good color matching	56 (94.9)
General color matching	3 (5.1)
Poor color matching	0 (0.0)
***Texture matching***	
Good texture matching	54 (91.5)
Average texture matching	5 (8.5)
Poor texture matching	0 (0.0)
***Sensory function recovery***	
S3 (good)	53 (89.8)
S2 (partial recovery)	6 (10.2)
S1 (slight recovery)	3 (5.1)
S0 (no recovery)	0 (0.0)
***Second stage operation***	
No need for secondary surgery	56 (94.9)
Skin flap volume reduction plastic surgery	3 (5.1)
Other cosmetic surgery	0 (0.0)

### Case Reports:

### Case one:

**Fig.1:**
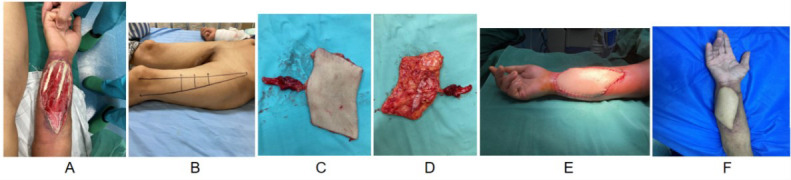
Female patient, 45 years old. Due to mechanical compression injury causing damage to the palmar tissue of the right forearm, the patient was admitted to the hospital for ALT perforator flap repair of the forearm defect. (A) Right forearm palmar tissue destruction with exposed tendon structure (range 10cm × 8cm); (B) Design and harvesting of a thigh anterior lateral chimeric perforator flap; (C&D) Appearance of femoral anterior lateral chimeric perforator and Flow through perforator flaps; (E) Immediate postoperative effect of skin flap surgery. A dual-lobed ALT flap was used in this case, with each segment aligned to accommodate the contour of the flexor tendon and maintain joint mobility; (F) After 16 months of follow-up, the color and texture were similar to the surrounding skin and soft tissue and the upper limb function was good.

A 45-year-old woman sustained a crush injury to the right forearm, resulting in an extensive soft tissue defect (10 × 8 cm) with exposure and rupture of the flexor tendons (Fig.1A). Initial debridement was performed, followed by immediate reconstruction using a free ALT perforator flap. The flap was designed with a dual-lobed structure to accommodate the palmar contour and tendon bed. A 10 cm pedicle length was preserved to allow tension-free anastomosis and optimal rotation (Fig.1B–D). Intraoperative assessment confirmed smooth microvascular flow without kinking (Fig.1E). By 16 months, the flap remained viable with supple texture, good color match, and functional restoration of finger flexion (Fig.1F).

### Case two:

**Fig.2:**
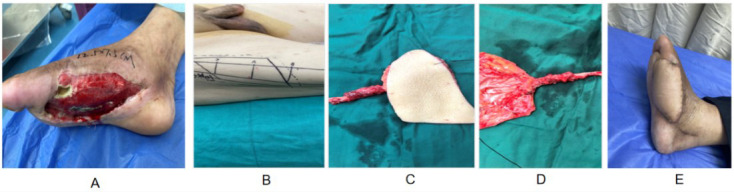
Male, 58 years old. (A) Right foot defect caused by agricultural machinery twisting injury to the heel of the right foot, with a defect range of 12.5cm × 7.5cm, reaching deep into the calcaneus and Achilles tendon; (B) Design and harvesting of the right ALT chimeric perforator flap; (C&D) Cut and free the skin flap. To match the curvature of the heel and reduce tension, a segmented flap layout was designed, with the vascular pedicle adjusted to avoid compression during weight-bearing; (E) There were no adverse reactions during the 12-month follow-up of the postoperative skin flap and the functional recovery was good.

Male, 58 years old. The right foot defect was caused by the twisting of the agricultural machinery on the heel of the right foot, with a defect range of 12.5cm × 7.5 cm, reaching deep into the calcaneus and Achilles tendon (Fig.2A). The ALT perforator flap repair surgery was performed. After debridement, a lateral thigh perforator flap was designed based on the location, shape and size of the soft tissue defect in the right foot and the position of the donor site (Fig.2B). The skin flap was cut and freed (Fig. 2C and D). There were no adverse reactions observed during the 12-month follow-up of the postoperative skin flap (Fig.2E). Normal weight-bearing and linear scars in the donor area were reported.

## DISCUSSION

In this study, clinical data of 62 patients with soft tissue limb defects who received ALT perforator flap treatment were analyzed. Of these, 59 patients reported complete skin flap survival, resulting in a survival rate of 95.2%, which is consistent with the previous studies.^8^-^10^ Chisolm et al.^10^ showed that the success rate of ultra-thin ALT-free flap in 16 cases was 100%.^10^ The ALT flap is based on the stability of the vascular anatomy of the lateral circumflex and descending cutaneous perforating branches of the thigh. The vascular pedicle can be extended to 8~12 cm and the diameter can reach 1.5~3.0 mm, which meets the needs of microsurgery.^8^-^11^ Wei et al.^12^ found that augmented reality technology has a sensitivity of 97.5% for locating the perforating branches of ALT skin flaps. This is primarily due to the precise repair of sensory nerves during surgery, where the lateral femoral cutaneous nerves are accurately sutured and aligned with the sensory nerves in the recipient area, promoting functional recovery and nerve nutrition.^13^,^14^

The anatomical characteristics of the blood supply artery innervation area of the ALT flap determine its suitability for repairing large-scale tissue defects.^10^ One of the key advantages of the ALT perforator flap lies in its capacity to balance aesthetic restoration with functional recovery.^11^ Through tailored flap thinning, alignment with natural skin tension lines, and the option of sensory nerve coaptation, the flap provides a pliable, color-matched coverage that does not restrict joint mobility or interfere with local biomechanics.^12^,^13^ This is particularly beneficial in reconstructions involving joints or exposed regions such as the hand, foot, or ankle, where both cosmetic and functional results are of paramount importance. In our case series, flap thickness and orientation were adjusted based on defect topography to optimize mobility and visual integration, demonstrating the clinical potential of the ALT flap in achieving aesthetic-functional synergy.^14^ In addition, studies have shown that the size of a single perforating blood supply area in the ALT anatomy is 10 cm × 6 cm. However, when adjacent perforating branches are combined, the area can be expanded to 20 cm × 10 cm.^15^,^16^ In this study, all sensory ALT perforator flaps achieved flap sensory recovery, which is similar to the previous reports.^14^-^17^ In addition, as one of the advantages of myocutaneous flaps, the most prominent feature of ALT perforator flaps is the selective use of perforating blood vessels while preserving the integrity of the donor muscle as much as possible, avoiding the risk of functional loss.^13^-^17^ The latest percutaneous dynamic infrared thermography technology has achieved an accuracy of 92.3% in locating the perforating branches of ALT skin flaps.^18^

The application of the multi-leaf design concept, thus, can be more widely applicable to different recipient area shapes.^19^,^20^ In clinical practice, the individualized design of ALT flaps often relies on the size, geometry, and anatomical curvature of the recipient defect, as well as the distribution of perforator vessels. In cases where the defect spans functionally sensitive areas—such as joints, palms, or heels—a multi-lobed design is adopted. This allows the flap to be divided into two or more segments with customized lengths, orientations, and vascular axes to better conform to the target region while preserving mobility and minimizing local tension. In our series, both Case-1 (Fig.1) and Case-2 (Fig.2) demonstrate this principle. For the palmar forearm defect in Case-1, a dual-lobed design was applied to accommodate tendon exposure and joint flexibility. In Case-2, covering the curved contour of the heel required a segmented flap with adjusted pedicle alignment to ensure secure coverage and prevent vascular compromise. These cases reflect the flexibility and adaptability of the ALT flap in reconstructive planning. Due to the ALT axial blood supply principle, based on perforating vessels, personalized remodeling can be achieved by considering the morphological characteristics of the recipient area during design.^18^-^20^ Moreover, the research on the use of ALT skin flaps for abdominal wall reconstruction.^21^,^22^ Three cases of flap inactivation were recorded in this article, with a complication rate of 4.8%. This is consistent with the range of 3% -8% reported in the literature.^4^,^22^,^23^ Flap necrosis remains a critical complication in ALT perforator flap surgery, often related to intraoperative vascular trauma, vasospasm, or postoperative mechanical compression.^4^,^23^ In our series, all three necrotic cases were retrospectively associated with these risk factors. To mitigate such outcomes, several preventive strategies were employed. First, careful microdissection of perforator vessels under magnification (×10) was conducted to minimize inadvertent trauma. Warm saline irrigation and topical papaverine were used when vasospasm was suspected. The flap inset was performed with attention to pedicle length and tension to avoid angulation or kinking. Postoperative protocols emphasized neutral limb positioning, limb elevation, and avoidance of joint flexion that could compromise vascular inflow or outflow. In the first 48 hours post-surgery, hourly flap monitoring was conducted to assess skin color, capillary refill, and Doppler signals. In two cases of partial or full-thickness necrosis, timely revision surgery with debridement and split-thickness skin grafting was performed, and pharmacological interventions including anticoagulants and vasodilators were administered. These measures collectively reduced the extent of tissue loss and preserved functional outcomes. However, the ALT flap still has some limitations. For example, the relatively thick texture of the flap may affect the effect of fine site reconstruction. Three patients in this group required secondary volume reduction plastic surgery, which highlights this issue. In addition, the anatomical variation of perforating vessels was substantial and the position of perforating vessels was not constant in approximately 10% to 15% of patients, which requires high microsurgical expertise from the operator.

Case-based reflections within our series offered practical insights for complication prevention and surgical refinement. In Case-5, we encountered intraoperative arterial vasospasm that significantly reduced perfusion. The vessel responded to topical papaverine and warm saline irrigation, but the incident highlighted the critical need for gentle dissection, minimal manipulation, and timely pharmacological intervention. In Case-7, distal flap edge necrosis was noted by day 3 postoperatively. A retrospective review suggested improper limb positioning contributed to venous congestion. Since then, we have adopted stricter postoperative positioning protocols—especially avoiding flexion near the pedicle—to maintain optimal flap perfusion. These examples underscore the importance of dynamically adapting intraoperative and postoperative strategies based on real-time surgical findings.

In our case series, favorable outcomes were closely associated with several intraoperative and perioperative factors. These included individualized flap design—especially the use of multi-lobed ALT flaps for complex geometries—tension-free flap inset, and thorough preoperative vascular mapping using Doppler ultrasound. Conversely, the three cases of flap necrosis appeared to share risk factors such as marginal perfusion deficits and suboptimal postoperative limb positioning. Early intervention through debridement and secondary grafting in these cases contributed to eventual wound healing. Based on these experiences, several surgical lessons were identified. First, meticulous microdissection and atraumatic handling of perforators are essential to maintaining flap viability. Second, intraoperative application of topical vasodilators and temperature-controlled irrigation helps prevent vasospasm. Third, proper limb positioning—ensuring the pedicle remains untwisted and decompressed—is crucial in the early postoperative period. These strategies are now consistently emphasized in our clinical protocol.

A more detailed examination of these three cases revealed that different mechanisms were involved. One case involved inadvertent injury to the dominant perforator vessel during dissection, leading to partial flap edge necrosis. Two other cases showed complete necrosis, which may have resulted from vasospasm of the small-caliber vessels due to prolonged manipulation and cold irrigation, as well as improper postoperative limb positioning that caused venous congestion. These complications highlight the multifactorial nature of flap failure. To minimize such risks, several intraoperative and postoperative measures should be adopted. During flap harvest, careful microdissection and preservation of multiple perforators are encouraged to ensure adequate perfusion redundancy. Avoiding excessive tension or torsion on the pedicle is essential during flap transfer. Intraoperative use of warm saline irrigation and topical vasodilators like papaverine can effectively reduce vasospasm risk. Postoperatively, maintaining neutral limb positioning, avoiding flexion at joints, elevating the limb, and performing regular flap perfusion assessments (especially within the first 48–72 hours) are vital to prevent vascular compromise and ensure flap survival.

The primary advantage of this study is its long-term follow-up, with an average duration of 32.9 ± 13.4 months. The results showed that the ALT perforator flap can be effectively used for soft tissue defects in limbs. However, the following points still need to be noted during surgery:

- The perforating status of the skin flap should be accurately explored before the surgery and the design of the skin flap should be based on the wound defect.
- There is a need to ensure that the vascular pedicle does not rotate or fold at sharp angles.
- Attention should be paid to reducing the ischemia time of the skin flap.
- There is a need to preserve multiple perforating branches during the surgery to facilitate the free combination of skin flaps.
- The size of the lobulated skin flap should be adjusted according to the thickness of the perforating branch to reduce the probability of insufficient blood supply to the flap.

This study presents several novel aspects that distinguish it from previously published ALT flap studies. First, it features a relatively large sample size (n=62) and an extended follow-up period (mean 32.9 ± 13.4 months), allowing for a more comprehensive understanding of long-term outcomes. Second, the study includes a dual focus on both aesthetic outcomes (color and texture matching) and functional recovery (sensory grading), offering a holistic view of postoperative success. Third, the practical application of individualized multi-lobed flap designs, based on vascular anatomy and wound morphology, demonstrates the adaptability of the ALT flap in diverse clinical scenarios. Future studies should consider conducting prospective, multicenter comparative trials to confirm these findings across varied populations and clinical indications. Incorporating objective functional assessment tools—such as grip strength, joint range of motion, and gait analysis—would enhance the robustness of clinical outcome evaluation. Furthermore, exploring the use of ALT flaps in high-risk or specialized patient populations, such as those with diabetic foot ulcers, oncologic defects, or pediatric limb injuries, may broaden the reconstructive applicability of this technique.

### Limitations:

This study, as a retrospective case series, has several inherent limitations. First, the heterogeneity among patients—including differences in defect location, size, comorbidities, and rehabilitation protocols—may affect the comparability of outcomes. Second, the absence of a control group precludes direct comparisons with other reconstructive techniques, limiting the ability to draw causal inferences. Third, the sample size was relatively small, which restricts the generalizability of our findings. Fourth, the follow-up duration varied considerably (8–60 months), and some patients had incomplete clinical documentation or shorter follow-up periods, which may have influenced the evaluation of long-term outcomes. To mitigate this, a minimum follow-up threshold of 8 months was applied, and subgroup analysis of patients with ≥24 months of follow-up showed consistent functional and aesthetic results. Fifth, the evaluation of functional recovery primarily relied on the MRC sensory grading system, which, although clinically useful, does not comprehensively reflect multidimensional limb function. This limitation is partly due to the retrospective nature of the study and variability in available follow-up data. Future studies should adopt prospective designs with larger sample sizes, standardized follow-up intervals (ideally beyond five years), and incorporate objective functional measures—such as grip strength, range of motion (ROM), gait analysis—as well as patient-reported outcome measures (PROMs), including visual analogue scales (VAS) or Likert-based satisfaction scores. These improvements would help establish a more robust, clinically relevant understanding of the long-term value of the ALT perforator flap.

## CONCLUSION

The long-term follow-up results of this study demonstrate that the ALT perforator flap can be effectively utilized as a reconstruction treatment plan for soft tissue defects in limbs, resulting in good functional recovery and aesthetic outcomes.

### Authors’ contributions:

**JH:** Study design, literature search and manuscript writing.

**CR and LH:** Data collection, data analysis and interpretation. Critical Review.

**JH:** Manuscript revision and validation and is responsible for the integrity of the study.

All authors have read and approved the final manuscript.

## References

[1] Goffinet L, Dantzer E (2020). Coverage of soft tissue defects in acute surgery for deep burns of the limbs. Ann Chir Plast Esthet.

[2] Hao Z, Tian S, Hu C, Jia Y (2022). Clinical application of retrograde sural neurofasciocutaneous flap repair combined with jingulian capsules to treat foot and ankle soft tissue defects. Pak J Med Sci.

[3] Cao ZM, Du W, Qing LM, Zhou ZB, Wu PF, Yu F (2019). Reconstructive surgery for foot and ankle defects in pediatric patients: Comparison between anterolateral thigh perforator flaps and deep inferior epigastric perforator flaps. Injury.

[4] Barnhill CW, Greyson MA, Iorio ML (2024). Superficial circumflex iliac artery perforator flap reconstruction of the upper extremity. Hand Clin.

[5] Song YG, Chen GZ, Song YL (1984). The free thigh flap: a new free flap concept based on the septocutaneous artery. Br J Plast Surg.

[6] Hsu CC, Loh CYY, Wei FC (2021). The Anterolateral Thigh Perforator Flap: Its Expanding Role in Lower Extremity Reconstruction. Clin Plast Surg.

[7] D'Arpa S, Colebunders B, Stillaert F, Monstrey S (2017). Pre-expanded Anterolateral Thigh Perforator Flap for Phalloplasty. Clin Plast Surg.

[8] Pang X, Wu P, Zhang X, Xiao Y, Pan D, Tang J (2021). Clinical application of polyfoliate anterolateral thigh perforator flap with single-perforator. Zhong Nan Da Xue Xue Bao Yi Xue Ban.

[9] Zhong L, He L, Yin D, Jin Z, Niu Y, Wang Z (2022). Application of anterolateral thigh bridge flap with free skin graft wrapping vascular bridge in complex calf soft tissue defects. Zhongguo Xiu Fu Chong Jian Wai Ke Za Zhi.

[10] Chisolm P, Dowd J, Zheng W, Wu E, Pierce M, Davidson B (2025). Feasibility of Super-thin Anterolateral Thigh Free Flap for Oral and Pharyngeal Reconstruction. Laryngoscope.

[11] Cang CW, Chen YX, Xian H, Zhao CY, Zhang JL, Zou QY (2022). Application of lobed anterolateral thigh chimeric perforator flap in repairing the complex soft tissue defects of limbs. Chin J Microsury.

[12] Wei B, Lu G, Bai Z, Osei-Hwedieh DO, Chen Y, Li Q (2025). Augmented reality in preoperative anterolateral thigh flap perforators positioning: A pilot diagnostic study. Oral Oncol.

[13] Ma C, Tian Z, Kalfarentzos E, Zhang Y, Zhang Z, Lam D (2016). Superficial circumflex iliac artery perforator flap for tongue reconstruction. Oral Surg Oral Med Oral Pathol Oral Radiol.

[14] Plonowska-Hirschfeld KA, House A, Park AM, Seth R, Heaton CM, Fridirici Z (2024). Increasing Pedicle Reach with Musculocutaneous Perforator Dissection in Anterolateral Thigh Free Flaps. Laryngoscope.

[15] Zhou P, Yang XD, Xu XD, Tang ML (2007). Morphological study and flap design of perforating arteries in the anterior lateral femoral region. Chin J Microsury.

[16] Wei FC, Jain V, Celik N, Chen HC, Chuang DC, Lin CH (2002). Have we found an ideal soft-tissue flap?An experience with 672 anterolateral thigh flaps. Plast Reconstr Surg.

[17] Pan Z, Zhao Y, Ye X, Wang J, Li X (2024). Surgical refinements and sensory and functional outcomes of using thinned sensate anterolateral thigh perforator flaps for foot and ankle reconstruction: A retrospective study. Medicine (Baltimore).

[18] Meier EL, De Jong T, Ulrich DJO, Hummelink S (2025). Preoperative perforator mapping of anterolateral thigh perforators via Projected Augmented Reality and Dynamic Infrared Thermography. J Plast Reconstr Aesthet Surg.

[19] Pedrazzi NE, Schweizer R, Klein HJ, Steiner S, Vonlanthen R, Hermanns T (2025). Versatility of the Anterolateral Thigh Flap for Abdominal Wall Reconstruction. Plast Reconstr Surg Glob Open.

[20] He J, Qing L, Wu P, Zhou Z, Yu F, Cao Z (2021). Individualized design of double skin paddle anterolateral thigh perforator flaps to repair complex soft tissue defects of the extremities: An anatomical study and retrospective cohort study. J Plast Reconstr Aesthet Surg.

[21] Yang Y, Cao ZM, Sun NZ, Qing LM, Wu PF, Tang JY (2024). Clinical effects of different types of flaps selected according to local conditions in the treatment of diabetic foot defects. J Orthop Surg Res.

[22] Scaglioni MF, Meroni M, Tomasetti PE, Rajan GP (2024). Head and neck reconstruction with the superficial circumflex iliac artery perforator (SCIP) free flap: Lessons learned after 73 cases. Head Neck.

[23] Di H, Yu Xia T, Ma C, Guo H, Xing P, Xia C (2024). Reconstruction of multiple long digital and hand defects using the multilobed anterolateral thigh perforator flap. Acta Orthop Traumatol Turc.

